# It ain’t what you do, it’s the way that you do it: The pitfalls of using routine data to measure early infant HIV diagnosis in HIV-exposed infants

**DOI:** 10.1371/journal.pone.0257496

**Published:** 2021-09-30

**Authors:** Elizabeth Chappell, Claire Thorne, Intira Jeannie Collins, Kathy Baisley, H. Manisha Yapa, Dickman Gareta, Till Bärnighausen, Kobus Herbst, Ali Judd

**Affiliations:** 1 MRC Clinical Trials Unit at UCL, London, United Kingdom; 2 UCL Great Ormond Street Institute of Child Health, London, United Kingdom; 3 MRC Tropical Epidemiology Group, London School of Hygiene & Tropical Medicine, London, United Kingdom; 4 Africa Health Research Institute, Mtubatuba, KwaZulu-Natal, South Africa; 5 The Kirby Institute University of New South Wales, Sydney, Australia; 6 Department of Global Health and Population, Harvard T.H. Chan School of Public Health, Boston, Massachusetts, United States of America; 7 Heidelberg Institute of Global Health, University of Heidelberg, Heidelberg, Germany; 8 Institute for Global Health UCL, Mortimer Market Centre, London, United Kingdom; 9 DSI-MRC South African Population Research Infrastructure Network (SAPRIN), Durban, KwaZulu-Natal, South Africa; University of Cape Town, SOUTH AFRICA

## Abstract

**Background:**

Early infant HIV diagnosis (EID) is critical to ensuring timely diagnosis of HIV-exposed infants, and treatment in those found to be infected. However estimates of coverage vary considerably, depending on data sources used. We used 4 methods to estimate coverage among a historical cohort of HIV-exposed infants in rural South Africa, between 2010–2016.

**Methods:**

We estimated the proportion of infants ever tested (methods 1–3) and tested by 7 weeks of age (1–4) as follows: (1) infants born to women identified as HIV-positive in demographic surveillance were linked to those with ≥1 EID result in routine laboratory surveillance; (2) the number of infants with ≥1 EID result in laboratory surveillance divided by the estimated number of HIV-exposed infants, calculated as total live births multiplied by antenatal HIV seroprevalence; (3) the number of infants with ≥1 EID result in routine laboratory surveillance, divided by the number of HIV-exposed infants as estimated by the district health service; (4) from documentation in infants’ Road-to-Health-booklets.

**Results:**

The proportion ever tested was 43%, 88% and 138% for methods 1–3, and by 7 weeks of age was 25%, 49%, 86% and 46% for methods 1–4 respectively.

**Conclusions:**

The four methods, applied to a range of routine data sources, resulted in estimates varying considerably, and the true coverage of EID remains unclear. Our findings highlight the importance of developing unique patient identifiers, improving training of healthcare providers using reporting systems, and ensuring the accuracy of healthcare records, to ensure the best possible health outcomes for HIV-exposed infants.

## Introduction

Since the launch of the prevention of mother-to-child transmission (PMTCT) program in South Africa in 2004, the rate of vertical transmission of Human Immunodeficiency Virus (HIV) in South Africa has fallen from 31.2% to 4.8% in 2017 [1]. Nonetheless, with a high antenatal HIV seroprevalence of >40% in some regions, a substantial number of infants are still born with HIV [2]. There is high early mortality in this group [3], making timely testing and diagnosis of these infants to enable linkage to care and treatment important, and the proportion of HIV-exposed infants who actually receive a PCR test a critical indicator of health systems performance.

Since 2004, South African national guidelines have recommended that all HIV-exposed infants receive early infant HIV diagnosis (EID) using deoxyribonucleic acid (DNA) polymerase chain reaction (PCR) tests by 6 weeks of age. These guidelines were updated in April 2015 to recommend a PCR test at birth, with a subsequent PCR test for confirmation for those testing positive, or a repeat test for those testing negative at 10–18 weeks of age [4]. Additional testing of symptomatic infants and at 6 weeks after the cessation of breastfeeding has been recommended throughout.

Previous estimates of the proportion of infants tested by 2 months of age in South Africa between 2010 and 2016 have ranged from 52% to over 100% [5–8]. Sources of data used by studies include patient records, national laboratory service data, aggregated reports from healthcare facilities and interview with caregivers. One study comparing national estimates from two sources of routinely collected data found substantial variation both between the two methods, as well as with each method when alternative definitions of the denominator of HIV-exposed infants (based either directly on the reported number of HIV-exposed infants, or on the total number of infants multiplied by the estimate antenatal HIV seroprevalence) were used [6]. The reasons for this variation and limitations of the sources of routinely collected data used are not always well investigated.

Given the importance of accurately estimating coverage in ensuring optimal care for this vulnerable population, we compare four different methods, using different sources of data, for estimating coverage among a historical cohort of infants born between 1^st^ June 2010 and 31^st^ December 2016 in the Hlabisa health sub-district, KwaZulu-Natal. We discuss the strengths, weaknesses and potential biases for each method, and make recommendations for improved monitoring.

## Materials and methods

The Hlabisa health sub-district lies within the uMkhanyakude district, KwaZulu-Natal, South Africa. It is a predominantly rural area, with a population of 228,000 people [9]. In 2016, the HIV prevalence was estimated at 48% among women attending antenatal care [10]. The sub-district contains 16 primary healthcare clinics and one hospital.

Since 2000, the Africa Health Research Institute (AHRI) has conducted demographic surveillance in part of the sub-district, which covers a population of 85,000 people [11]. Households and individuals are followed longitudinally, and dried blood spots for HIV surveillance are collected on resident adults aged ≥15 years [9]. Overall participation has been estimated at >99% [11], with 77% of adults estimated to have participated in the HIV surveillance component at least once in the first 9 years of becoming resident [12]. Participating individuals are linked to records in Three Integrated Electronic Register (TIER).net, the national Department of Health antiretroviral therapy (ART) monitoring system, from the 17 healthcare facilities in the sub-district, using a deterministic and probabilistic algorithm based on South African national ID number, name, cell phone number, date of birth, sex and nearest clinic.

The National Health Laboratory Service (NHLS) is responsible for laboratory testing in public facilities in South Africa. Data on all HIV DNA PCR tests conducted (regardless of the age of the individual) between 1^st^ June 2010 and 1^st^ July 2017 were downloaded from the NHLS database through a secure file transfer protocol. A second deterministic and probabilistic data linkage algorithm, based on first name, surname, sex, date of birth, facility at which the test was conducted and the infant’s facility ID was used to identify repeat tests on the same child. Although South African national ID number is a field on the test request forms, it was completed for <1% of PCR tests (among which no two records had the same ID number recorded), and so was not used for linkage. To assess the accuracy of this algorithm the proportion of individuals in the resulting deduplicated dataset who had the same surname, date of birth and sex as another individual was compared to the same proportion in a dataset of known unique individuals from the AHRI surveillance area.

The four methods used to estimate testing coverage are described below and in Table 1.

**Table 1:** Summary of the four methods used to estimate PCR testing coverage.

	Method 1: NHLS-AHRI surveillance	Method 2: NHLS-SSA/ANCHSS	Method 3: NHLS-DHIS	Method 4: Road-to-Health booklets (MONARCH)
Numerator (number of HIV-exposed infants who received a PCR test)	Infants with a linked NHLS PCR test	Number of infants with a PCR test in the NHLS database	Number of infants with a PCR test in the NHLS database	Infants with a PCR test recorded in their Road-to-Health booklet from birth to 6-week immunisation visit
Denominator (number of HIV-exposed infants)	Infants born to women testing positive in serosurvey or initiating ART in TIER.net database	Number of live births from SSA (adjusted for late registrations) multiplied by antenatal HIV seroprevalence from ANCHSS (adjusted for incident HIV between sampling and delivery)	Aggregated returns of number of live births to HIV-positive women sent from clinics to DHIS	Infants born to HIV-positive women attending antenatal care at one of 7 clinics in the AHRI surveillance area
Linked numerator and denominator?	Yes	No	No	Yes
Geographical area covered	AHRI surveillance area	Hlabisa health sub-district	Hlabisa health sub-district	AHRI surveillance area
Calendar time period covered (infant’s date of birth)	June 2010 to December 2016	June 2010 to December 2016	April 2014 to December 2016	July 2015 to December 2016

AHRI: Africa Health Research Institute; ANCHSS: National Antenatal Sentinel HIV and Syphilis Survey Report; DHIS: District Health Information System; HIV: Human Immunodeficiency Virus; MONARCH: Management and Optimization of Nutrition, Antenatal, Reproductive, Child Health and HIV Care; NHLS: National Health Laboratory Service; PCR: Polymerase Chain Reaction; SSA: Statistics South Africa.

### Method 1: NHLS-AHRI surveillance

Infants born in the AHRI surveillance area whose mothers were identified as HIV-positive through participation in HIV surveillance and/or linkage to TIER.net were the denominator. A third deterministic and probabilistic linkage algorithm between these infants and the NHLS database (based on infant’s first name, surname, date of birth and sex) was used to estimate the proportion tested.

### Method 2: NHLS-Statistics South Africa (SSA)/National Antenatal Sentinel HIV and Syphilis Survey Report (ANCHSS)

Testing coverage was estimated using the number of infants tested in the NHLS database, divided by the number of HIV-exposed infants born in the sub-district, which was calculated for each year of birth using SSA estimates of the number of live births multiplied by ANCHSS estimates of the antenatal HIV seroprevalence.

SSA publishes the number of live births annually, using data on birth registrations [13–19], however late registration of some births means that reported estimates for each year of birth increase over time. We used the average increase in reported estimates in previous years to adjust estimates for more recent calendar years to reflect this (see S1 Table). Figures are published on a district level, so were scaled for the sub-district using the size of the population of the Hlabisa sub-district and the uMkhanyakude district (228,000/625,000 = 36.5%) [9,20].

Estimates of antenatal seroprevalence were based on those from the annual ANCHSS survey [21], for which consenting women attending antenatal care at a sample of public healthcare facilities across South Africa receive an HIV antibody test. We adjusted estimates to account for HIV infections acquired between sampling for the survey and delivery which would not have been captured, with the rate of incident HIV during pregnancy in the sub-district estimated at 4.5 per 100-person-years [22]. Between 2010 and 2014 only women at their first antenatal clinic visit were included in the ANCHSS survey, which occurs at a median gestational age of 19.5 weeks in the sub-district (estimated using data from MONARCH, see below). From 2015, ANCHSS methods were updated to remove this restriction, resulting in an estimated median gestational age of 37.5 weeks at sampling.

For comparison, testing coverage based on unadjusted estimates of the number of live births and antenatal seroprevalence, and PCR test data prior to deduplication of repeat tests, was also calculated.

### Method 3: NHLS-District Health Information System (DHIS)

For this method, the coverage estimate was derived from the number of infants tested (NHLS data), divided by an estimate of the number of HIV-exposed infants born in the sub-district from DHIS data. All public health facilities in South Africa send aggregated data on their activity to DHIS, including the number of live births to women with HIV. These estimates were adjusted to account for infants not born in healthcare facilities, estimated through AHRI surveillance to be 3% of all births. DHIS data were only available from April 2014 onwards.

### Method 4: Road-to-Health booklets

This method used routinely collected data which was accessed through MONARCH, a trial which evaluated the impact of a quality improvement intervention package on PMTCT processes [23,24]. All children in South Africa are given a patient-held medical record at birth, called the Road-to-Health Booklet, in which information including HIV testing is recorded. As part of the trial, booklets belonging to all infants born to women receiving antenatal care at the 7 clinics in the AHRI surveillance area between July 2015 and December 2016 were photographed up to the 6-week postnatal visit. By 6 weeks of age only 25% of infants in MONARCH had had their 6-week postnatal visit, compared to 90% by 7 weeks of age. Testing coverage was estimated as the proportion of HIV-exposed infants with a PCR test recorded. MONARCH was conducted after the change to testing guidelines, so all infants would be expected to be tested at birth, but a sensitivity analysis was conducted to compare coverage among infants whose booklet was photographed at their 6-week visit to that in infants whose booklet was only seen earlier using a chi-squared test with Rao-Scott correction to account for clustering by clinic.

### Estimation of testing coverage

As data on PCR testing were only available to the 6-week postnatal visit for MONARCH, estimates of the overall testing coverage (proportion of infants ever tested) were compared across methods 1–3 only, and estimates of testing coverage by 7 weeks of age were compared across all four methods. Coverage was estimated by year of birth, and separately before and after the introduction of birth testing in the national guidelines (<1^st^ April 2015 vs. ≥1^st^ April 2015).

### Ethical approval

Ethical approval for: (i) the demographic surveillance, linkage to TIER.net, NHLS and other routine data sources, and analyses of these data; and (ii) the MONARCH trial; were granted by the University of KwaZulu-Natal Biomedical Research Ethics Committee (references BE290/16 and BE209/14 respectively). A waiver of the requirement for individual level informed consent was awarded, given the use of routine Department of Health data. Directly identifiable variables were required for the purposes of data linkage and were accessed in an on-premise secure environment.

## Results

**Fig 1:**
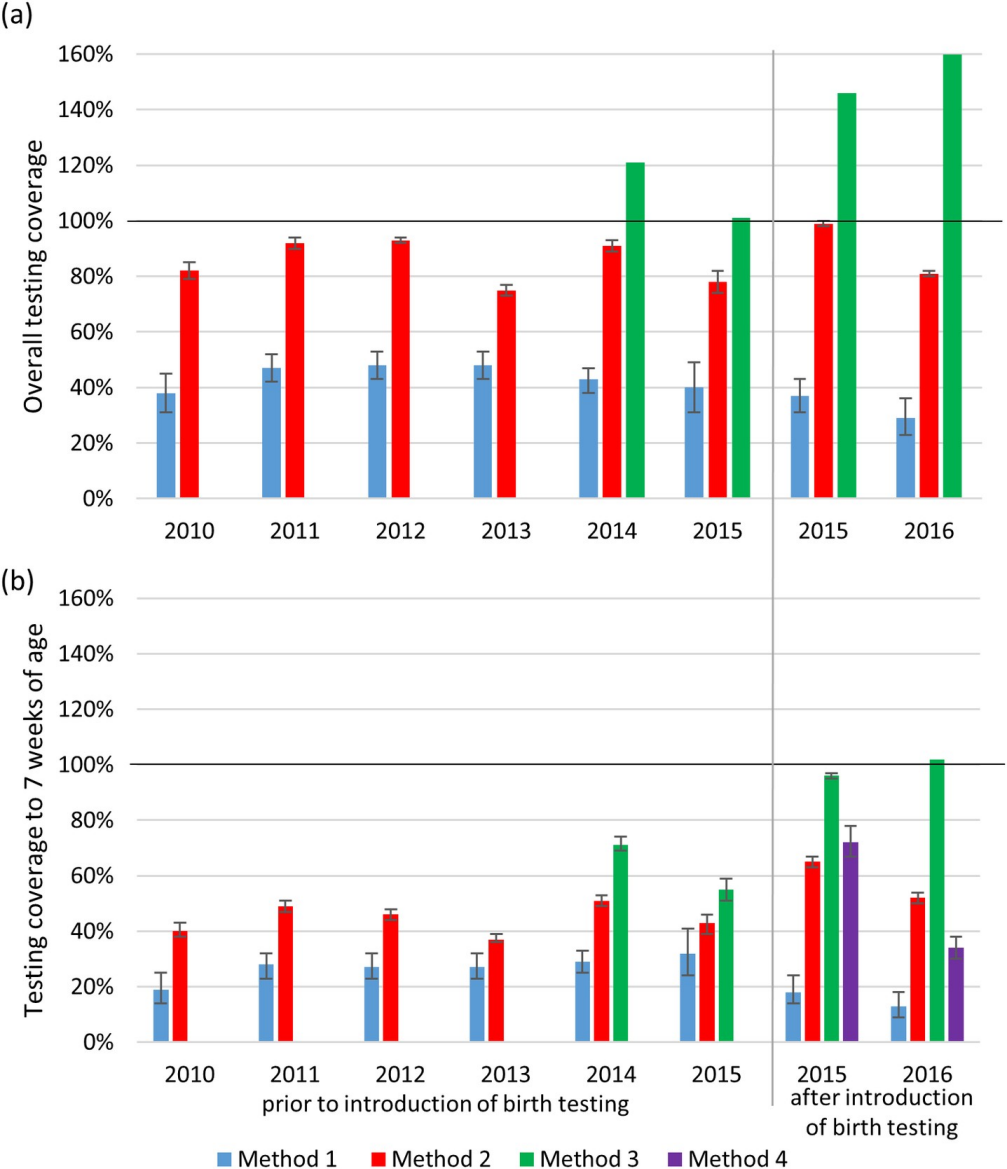
Comparison of estimates of coverage, both (a) overall, and (b) to 7 weeks of age. Data only available from April 2014 onwards for Method 3 and from July 2015 onwards for Method 4. Method 1 = NHLS-AHRI surveillance, Method 2 = NHLS-SSA/ANCHSS, Method 3 = NHLS-DHIS, Method 4 = Road-to-Health booklets (MONARCH). AHRI: Africa Health Research Institute; ANCHSS: National Antenatal Sentinel HIV and Syphilis Survey Report; DHIS: District Health Information System; NHLS: National Health Laboratory Service; SSA: Statistics South Africa.

The estimates of testing coverage in infants born to women living with HIV from each of the four methods are shown in Table 2 and Fig 1. Details of the calculations for each method are shown in S1–S5 Tables.

**Table 2:** Comparison of estimates of testing coverage.

Guideline time period	Calendar year of birth	Overall testing coverage according to method:			Testing coverage to 7 weeks of age according to method:			
		1: NHLS-AHRI surveillance	2: NHLS-SSA/ ANCHSS	3: NHLS-DHIS	1: NHLS-AHRI surveillance	2: NHLS-SSA/ ANCHSS	3: NHLS-DHIS	4: Road-to-Health booklets (MONARCH)
		n/N (%) {95% confidence interval}						
Prior to introduction of birth testing	June—December 2010	73/192 (38%) {31%, 45%}	1,044/1,280 (82%) {79%, 84%}	-	37/192 (19%) {14%, 25%}	517/1,280 (40%) {38%, 43%}	-	-
	2011	183/386 (47%) {42%, 52%}	2,084/2,275 (92%) {90%, 93%}	-	107/386 (28%) {23%, 32%}	1,119/2,275 (49%) {47%, 51%}	-	-
	2012	164/342 (48%) {43%, 53%}	2,234/2,400 (93%) {92%, 94%}	-	93/342 (27%) {23%, 32%}	1,102/2,400 (46%) {44%, 48%}	-	-
	2013	167/349 (48%) {43%, 53%}	2,173/2,898 (75%) {73%, 77%}	-	94/349 (27%) {23%, 32%}	1,081/2,898 (37%) {36%, 39%}	-	-
	2014	187/437 (43%) {38%, 47%}	2,373/2,620 (91%) {89%, 92%}	1,737/1,438 (121%) {-}	126/437 (29%) {25%, 33%}	1,329/2,620 (51%) {49%, 53%}	1,026/1,438 (71%) {69%, 74%}	-
	January—March 2015	48/121 (40%) {31%, 49%}	582/750 (78%) {74%, 81%}	582/578 (101%) {-}	39/121 (32%) {24%, 41%}	320/750 (43%) {39%, 46%}	320/578 (55%) {51%, 59%}	-
After introduction of birth testing	April—December 2015	89/240 (37%) {31%, 43%}	2,221/2,250 (99%) {98%, 99%}	2,221/1,521 (146%) {-}	44/240 (18%) {14%, 24%}	1,463/2,250 (65%) {63%, 67%}	1,463/1,521 (96%) {95%, 97%}	192/265 (72%) {67%, 78%}
	2016	54/187 (29%) {23%, 36%}	2,523/3,097 (81%) {80%, 83%}	2,523/1,578 (160%) {-}	24/187 (13%) {9%, 18%}	1,605/3,097 (52%) {50%, 54%}	1,605/1,578 (102%) {-}	186/548 (34%) {30%, 38%}
Overall		965/2,254 (43%) {41%, 45%}	15,234/17,570 (87%) {86%, 87%}	7,063/5,115 (138%) {-}	564/2,254 (25%) {23%, 27%}	8,536/17,570 (49%) {48%, 49%}	4,414/5,115 (86%) {85%, 87%}	378/813 (46%) {42%, 49%}

Data only available from April 2014 onwards for Method 3 and from July 2015 onwards for Method 4. AHRI: Africa Health Research Institute; ANCHSS: National Antenatal Sentinel HIV and Syphilis Survey Report; DHIS: District Health Information System; NHLS: National Health Laboratory Service; SSA: Statistics South Africa. Confidence intervals are not presented where the estimated proportion exceeds 100%.

In total, 17,622 PCR tests were extracted from the NHLS database, and following deduplication of those with repeat tests, 15,234 unique infants were identified. The proportion of these infants who had the same surname, date of birth and sex as another infant was 5.7%, compared to 4% in a dataset of known unique individuals.

For method 1, 2,254 HIV-exposed infants were identified, of whom 965 were linked to a PCR test in the NHLS database, giving an overall testing coverage of 43%. By year, this increased from 38% in 2010 to 48% in 2012 and 2013, before falling again to 29% in 2016.

With method 2, the total number of HIV-exposed infants born within the sub-district was estimated at 17,570, giving a resulting overall testing coverage estimate across the whole time period of 87% (15,234/17,570). By year of birth, estimates ranged from 75% to 99%, with no clear trend over time. The use of unadjusted estimates in the calculation of the number of HIV-exposed infants and of PCR test data prior to deduplication resulted in an increase in the estimate of testing coverage over the whole time period from 88% to 109%.

Using method 3, the number of infants born within the sub-district from April 2014 was estimated to be 5,115, with the corresponding number of infants tested being higher at 7,063. Testing coverage for this time period therefore impossibly exceeded 100%, at 138%, ranging from 101% to 160% by year of birth.

Comparing estimates of coverage to 7 weeks of age across methods 1–3, the same pattern was observed as for overall coverage, with the lowest estimate coming from method 1 and the highest from method 3. For method 4, 813 infants included in the MONARCH trial had available data recorded from their Road-to-Health booklet, of whom 290 (35%) had their booklet photographed at their 6-week visit compared to 547 (65%) whose booklet was only seen at earlier visits. In total, 378 had a PCR test recorded, giving an estimate of testing coverage to 7 weeks of age of 46%, somewhere in the middle of those made with the other methods. There was evidence that the proportion of infants with PCR test data recorded was higher among those seen at 6 weeks compared to those not (147/290 (51%) vs. 231/523 (44%), p = 0.039).

## Discussion

In this analysis we have explored the use of different methods and sources of routinely collected data in estimating the proportion of HIV-exposed infants in a rural South African setting with high antenatal HIV prevalence who received a PCR test. There was high variation in the estimates from each method.

A summary of the limitations of each method is shown in Table 3. Methods 1 to 3 relied on accurate linkage of repeat tests on the same infant within NHLS. It is known that an infant’s mother’s name may be used on the laboratory test request form instead of the infant’s for tests conducted in early life (as their name may not yet have been chosen), which makes linkage to subsequent tests difficult. The lack of a unique identifier also made deterministic linkage difficult. Although South African national ID is often missing on NHLS forms in general, recording is particularly poor for test requests for infants; ID numbers are only allocated at civil registration of birth, and so many infants don’t have a number at the time of testing. The proportion of infants in our final ‘deduplicated’ dataset who had the same surname, date of birth and sex was slightly higher than observed in another dataset of known unique individuals, suggesting a small amount of underlinkage. Underlinkage of repeat tests would have resulted in overestimation of the total number of unique infants tested, and thus overestimation of coverage. Conversely, limitations of the available identifiers may have also resulted in missed links between demographic surveillance and NHLS data in method 1, leading to underestimation of testing coverage; this may explain why estimates based on method 1 were lower than those for the other methods. Another limitation of method 1 is that only women who agreed to participate in HIV surveillance or were on treatment would be identified. Poor maternal adherence to treatment is known to be associated with poor uptake of EID services [25], so testing coverage among the infants of women not captured here may be lower.

**Table 3:** Limitations of the methods and the likely direction of bias on the estimate of coverage.

	Method	Data source	Issue	Type of error on numerator/ denominator	Likely direction of bias on estimate of coverage	Adjustment made
Numerator(PCR tests)	1, 2, 3	NHLS PCR dataset	NHLS data limitations, including use of mother’s name instead of infant’s and lack of unique identifier, may have led to failure to identify repeat tests on the same infant	Misclassification	Overestimation	
	1	NHLS-AHRI surveillance	Failure to link infants to their PCR tests	Misclassification	Underestimation	
	4	Recording of PCR tests in Road-to-Health booklets	Known poor completion of PCR data in Road-to-health booklet	Misclassification	Underestimation	
			Not all infants had their Road-to-health booklet photographed at their 6 week visit	Selection bias	Underestimation	
Denominator (HIV-exposed infants)	1	Women in AHRI surveillance known to have HIV	Only women on ART or willing to participate in serosurvey can be included	Selection bias	Either	
			Small number of women included	Reliability	Either	
	2	SSA/ANCHSS	Seroprevalence estimate based on small number of survey participants in each district	Reliability	Either	
			Women acquiring HIV after sampling for survey not included	Misclassification	Overestimation	Estimate of incident HIV during pregnancy used to adjust seroprevalence
			Underestimation of number births due to late registration	Misclassification	Overestimation	Change in reported numbers over time used to adjust estimates for more recent years
			Numerator not directly linked to denominator	Validity	Either	
	3	DHIS	Known underreporting of births in DHIS data	Misclassification	Overestimation	
			Infants born outside of healthcare facility not included	Misclassification	Overestimation	Numbers adjusted for infants born at home
			Numerator not directly linked to denominator	Validity	Either	

AHRI: Africa Health Research Institute; ANCHSS: National Antenatal Sentinel HIV and Syphilis Survey Report; DHIS: District Health Information System; HIV: Human Immunodeficiency Virus; MONARCH: Management and Optimization of Nutrition, Antenatal, Reproductive, Child Health and HIV Care; NHLS: National Health Laboratory Service; PCR: Polymerase Chain Reaction; SSA: Statistics South Africa.

For method 2, based on data from SSA and the ANCHSS, accurately estimating the number of HIV-exposed infants was difficult. Reported seroprevalence from ANCHSS varied substantially by year, for example from 41% to 35% to 44% in 2011, 2012 and 2013 respectively, resulting in corresponding variation in testing coverage estimates over time. This variation is likely due to small numbers sampled in each district, for example only 361 women from 20 clinics in uMkhanyakude in 2015 [21]. Similarly, it was unclear whether we fully accounted for late registrations of births; adjustments were made using known underreporting by 5 years of age, though completeness of birth registration in South Africa was only estimated at 90% by this age for those born in 2008 [26].

Estimates made using data from DHIS (method 3) were clearly incorrect as they exceeded 100% for all calendar years. This suggests significant underestimation of the total number of HIV-exposed infants born in the sub-district, with reported numbers being less than half of those calculated in method 2. DHIS does have data quality assessment processes, but other studies have reported unreliable data collection, with errors arising from poorly designed data collection tools, incorrect transcription of values, and staff not knowing the definition of all data items required [6,27].

Method 4 estimates, based on Road-to-Health booklets, were lower than those from methods 3, and similar to those from method 2. A cross-sectional study in Pretoria, South Africa in 2012 found that only 67% of HIV-exposed infants had the 6 week PCR test recorded in their booklet, and that 24% had no record of maternal HIV status [28]. It is known that parents often request HIV-related data be omitted from booklets or remove relevant pages themselves, to protect confidentiality if the booklet is seen by other family members or school staff, and therefore may not reflect actual EID access [29]. It is possible that recording of information in Road-to-Health booklets may have been more complete than normal here, given that facilities knew data were being used for a research study.

Given the limitations to each of the methods described above, apart from method 3 which produced estimates over 100% for some time period and thus may be considered the least reliable, it is unclear which method should be considered most accurate. The use of aggregated or population-level data impacts validity, as different infants may be included in the numerator and denominator, and thus the use of individual-level data with methods 1 and 4 may therefore be preferable. It should however be noted that the use of widely available and routinely collected data for methods 2 and 3 enables quicker and easier analysis, which may be more easily scaled up to estimate coverage in a larger population.

Three other South African studies have used approaches similar to method 2 to estimate EID coverage, however none deduplicated infants with repeat tests in the NHLS data or made adjustments to the number of live births and antenatal seroprevalence. Estimates from these studies ranged from 52–94% [5,6,27], and were higher than the adjusted estimates reported here (but closer to the unadjusted estimates presented in the supplementary tables), demonstrating overestimation caused by inability to account for limitations of data. Other South African studies assessing EID coverage have used electronic health records from single hospitals, where identification of HIV-exposed infants and of repeat tests is likely to have been a simpler process than in our study across healthcare clinics. Kalk et al looked at coverage at a healthcare facility in the Western Cape between February 2014 and June 2016, with 89% of HIV-exposed infants ever reported to have received a PCR test [7]. Smith et al estimated EID coverage at a hospital in uMkhanyakude outside of Hlabisa sub-district to be 54% in 2012 [30]. Finally, UNAIDS modelled estimates of EID coverage in South Africa were higher than those here, but varied substantially year-on-year, for example from 114% in 2015 to 79% in 2016 to 101% in 2017, further highlighting the methodological difficulties in estimating this indicator [31].

Our findings lead to several recommendations for improvements to policy and practice, which may have a public health benefit beyond EID. Firstly, the more widespread use of a unique patient identifier should be encouraged to enable accurate identification of repeat tests on the same infant and linkage to other data sources. Earlier assignment of the national ID number or the use of a number specific to the laboratory service should be considered. This would also facilitate tracing and follow-up of infants who move clinics after diagnosis. The national implementation of the Health Patient Registration System (HPRS) is working to improve use of ID numbers in South Africa [32]. Secondly, reasons for the current poor completion of DHIS tools need to be better understood, and suitable training and support procedures identified, with the importance of their use as a tool for monitoring healthcare systems emphasised. Thirdly, alternative ways of maintaining accurate and complete records of patient data while mitigating concerns about privacy violations should be explored, for example, using codes in Road-to-Health booklets to record HIV-related information [28].

Despite access to multiple sources of data, and application of a variety of methods, the true level of PCR testing coverage in the Hlabisa health sub-district cannot be confidently estimated. It is critical to ensure that infants at risk of HIV are appropriately followed and tested after birth, to eliminate the risk of infections remaining undiagnosed and enable early initiation of ART. While the use of routine data from sources such as those used here is increasing, both in South Africa and across other settings, few studies have explored their limitations or the impact of these limitations on results in this detail. There are limitations to all sources of routinely collected data, which should be considered when interpreting estimates, as well as for informing future improvements to data collection systems and processes. Improved recording of test data in the Road-to-Health booklets and laboratory forms, along with more widespread use of a unique patient identifier, is required, both for quality and continuity of clinical care to ensure the best possible outcomes, and surveillance and research purposes.

## Supporting information

S1 Table Adjustment of number of live births as reported by SSA (for method 2). (DOCX)

S2 Table Calculation of testing coverage estimates using method 1 (NHLS-AHRI surveillance). (DOCX)

S3 Table Calculation of testing coverage estimates using method 2 (NHLS-SSA/ANCHSS). (DOCX)

S4 Table Calculation of testing coverage estimates using method 3 (NHLS-DHIS). (DOCX)

S5 Table Calculation of testing coverage estimates using method 4 (road-to-health-booklets). (DOCX)
