# Supplementary material for: It ain’t what you do, it’s the way that you do it: The pitfalls of using routine data to measure early infant HIV diagnosis in HIV-exposed infants
Source: PLoS One. 2021 Sep 30;16(9):e0257496. doi: 10.1371/journal.pone.0257496 (PMC8483382; doi:10.1371/journal.pone.0257496)
Supplement: S1 Table — (DOCX) [file pone.0257496.s001.docx]

**Supplementary Table 1: Adjustment of number of live births as reported by SSA (for method 2)**

| Calendar year of birth | Initially reported estimate | Most recently reported estimate | Years between initial and most recent estimates | Adjustment of most recent estimate | Adjusted estimate | Increase from most recently reported to adjusted estimate |
| --- | --- | --- | --- | --- | --- | --- |
| Source of data | SSA | SSA |  | * |  |  |
| Method of calculation | A | B |  | C | D=B*C | D-B |
| 2010 | 10,592 | 14,013 | 5 | - | 14,013 | 0 |
| 2011 | 11,107 | 14,785 | 4 | x (1.323/1.322) | 14,796 | 11 |
| 2012 | 11,722 | 18,088 | 4 | x (1.323/1.322) | 18,102 | 14 |
| 2013 | 13,231 | 17,474 | 3 | x (1.323/1.312) | 17,621 | 147 |
| 2014 | 13,353 | 16,738 | 2 | x (1.323/1.263) | 17,533 | 795 |
| 2015 | 12,992 | 16,340 | 1 | x (1.323/1.217) | 17,763 | 1,423 |
| 2016 | 13,858 | 13,858 | 0 | x 1.323 | 18,334 | 4,476 |

SSA: Statistics South Africa.

*For example, the adjustment made to the estimate for 2014 was as follows: The most recent estimate of the number of births in 2014 came from the 2016 report, 2 years after the initial estimate. The adjusted estimate was therefore calculated by first dividing by 1.263 (as the average percentage increase between the initial estimate and the one 2 years later was 26.3%), and then multiplying by 1.323 (as the average percentage increase from the initial report to the one 5 years later, our best estimate of the true figure, was 32.3%). This gave a final adjusted estimate of 17,533 births in 2014, an increase of 795 over the most recently reported estimate for this year.

|  | Years since initial estimate | | | | | |
| --- | --- | --- | --- | --- | --- | --- |
|  | 0 | 1 | 2 | 3 | 4 | 5 |
| Average increase in reported number of live births | 0.0% | 21.7% | 26.3% | 31.2% | 32.2% | 32.3% |
