# Supplementary material for: It ain’t what you do, it’s the way that you do it: The pitfalls of using routine data to measure early infant HIV diagnosis in HIV-exposed infants
Source: PLoS One. 2021 Sep 30;16(9):e0257496. doi: 10.1371/journal.pone.0257496 (PMC8483382; doi:10.1371/journal.pone.0257496)
Supplement: S2 Table — (DOCX) [file pone.0257496.s002.docx]

**Supplementary Table 2: Calculation of testing coverage estimates using method 1 (NHLS-AHRI surveillance)**

| Guideline time period | Calendar year of birth | Number of HIV-exposed infants identified | Number with a linked PCR test | Number with a linked PCR test by 7 weeks of age | **Overall testing coverage** | **Testing coverage to 7 weeks of age** |
| --- | --- | --- | --- | --- | --- | --- |
| Source of data | | AHRI surveillance | NHLS | NHLS |  |  |
| Method of calculation | | A | B | C | **B/A** | **C/A** |
| Prior to introduction of birth testing | June - December 2010 | 192 | 73 | 37 | **38%** | **19%** |
|  | 2011 | 386 | 183 | 107 | **47%** | **28%** |
|  | 2012 | 342 | 164 | 93 | **48%** | **27%** |
|  | 2013 | 349 | 167 | 94 | **48%** | **27%** |
|  | 2014 | 437 | 187 | 126 | **43%** | **29%** |
|  | January - March 2015 | 121 | 48 | 39 | **40%** | **32%** |
|  | Total | 1,827 | 822 | 496 | **45%** | **27%** |
| After introduction of birth testing | April - December 2015 | 240 | 89 | 44 | **37%** | **18%** |
|  | 2016 | 187 | 54 | 24 | **29%** | **13%** |
|  | Total | 427 | 143 | 68 | **33%** | **16%** |
| TOTAL | | 2,254 | 965 | 564 | **43%** | **25%** |

AHRI: Africa Health Research Institute; HIV: Human Immunodeficiency Virus; NHLS: National Health Laboratory Service; PCR: Polymerase Chain Reaction.
