# Supplementary material for: It ain’t what you do, it’s the way that you do it: The pitfalls of using routine data to measure early infant HIV diagnosis in HIV-exposed infants
Source: PLoS One. 2021 Sep 30;16(9):e0257496. doi: 10.1371/journal.pone.0257496 (PMC8483382; doi:10.1371/journal.pone.0257496)
Supplement: S3 Table — (DOCX) [file pone.0257496.s003.docx]

**Supplementary Table 3: Calculation of testing coverage estimates using method 2 (NHLS-SSA/ANCHSS)**

| Guideline time period | Calendar year of birth | Number of live births in uMkhanyakude in whole year, from SSA 2016 report | Adjusted number of live births | Antenatal seroprevalence, as reported by ANCHSS | Adjusted antenatal seroprevalence, to reflect incident HIV between sampling and delivery | Population of Hlabisa sub-district as proportion of uMkhanyakude | Time (years) | Number of HIV-exposed infants born in Hlabisa sub-district | Number of infants tested | Number of infants tested by 7 weeks of age | **Overall testing coverage** | **Testing coverage to 7 weeks of age** | Number of HIV-exposed infants born in Hlabisa sub-district  - unadjusted estimate | Number of infants tested  - prior to deduplication | Number of infants tested by 7 weeks of age  - prior to deduplication | **Overall testing coverage**  **- unadjusted estimate** | **Testing coverage to 7 weeks of age**  **- unadjusted estimate** |
| --- | --- | --- | --- | --- | --- | --- | --- | --- | --- | --- | --- | --- | --- | --- | --- | --- | --- |
| Source of data | | [17] |  | [25] |  | [11, 24] |  |  | NHLS | NHLS |  |  |  |  |  |  |  |
| Method of calculation | | A | B=see ST5 | C | D# | E | F | G=B*D  *E*F | H | I | **H/G** | **I/G** | J=A*C  *E*F | K | L | **K/J** | **L/J** |
| Prior to introduction of birth testing | June - December 2010 | 14,013 | 14,013 | 41.9% | 42.9% | 36.5% | 7/12 | 1,280 | 1,044 | 517 | **82%** | **40%** | 1,249 | 1,181 | 522 | **95%** | **42%** |
|  | 2011 | 14,785 | 14,796 | 41.1% | 42.1% | 36.5% | 1 | 2,275 | 2,084 | 1,119 | **92%** | **49%** | 2,217 | 2,408 | 1,123 | **109%** | **51%** |
|  | 2012 | 18,088 | 18,102 | 35.2% | 36.4% | 36.5% | 1 | 2,400 | 2,234 | 1,102 | **93%** | **46%** | 2,323 | 2,569 | 1,109 | **111%** | **48%** |
|  | 2013 | 17,474 | 17,621 | 44.1% | 45.1% | 36.5% | 1 | 2,898 | 2,173 | 1,081 | **75%** | **37%** | 2,811 | 2,450 | 1,084 | **87%** | **39%** |
|  | 2014 | 16,738 | 17,533 | 39.9% | 41.0% | 36.5% | 1 | 2,620 | 2,373 | 1,329 | **91%** | **51%** | 2,436 | 2,667 | 1,332 | **109%** | **55%** |
|  | January - March 2015 | 16,340 | 17,763 | 46.3% | 46.3% | 36.5% | 3/12 | 750 | 582 | 320 | **78%** | **43%** | 690 | 650 | 321 | **94%** | **47%** |
|  | Total |  |  |  |  |  |  | 12,223 | 10,490 | 5,468 | **86%** | **45%** | 11,726 | 11,925 | 5,491 | **102%** | **47%** |
| After introduction of birth testing | April - December 2015 | 16,340 | 17,763 | 46.3% | 46.3% | 36.5% | 9/12 | 2,250 | 2,221 | 1,463 | **99%** | **65%** | 2,070 | 2,678 | 1,524 | **129%** | **74%** |
|  | 2016 | 13,858 | 18,334 | 46.3% | 46.3% | 36.5% | 1 | 3,097 | 2,523 | 1,605 | **81%** | **52%** | 2,341 | 3,019 | 1,658 | **129%** | **71%** |
|  | Total |  |  |  |  |  |  | 5,347 | 4,744 | 3,068 | **89%** | **57%** | 4,411 | 5,697 | 3,182 | **129%** | **72%** |
| TOTAL | |  |  |  |  |  |  | 17,570 | 15,234 | 8,536 | **87%** | **49%** | 16,137 | 17,622 | 8,673 | **109%** | **54%** |

#D=(1-C)*(risk of HIV per week)*(40-median gestational age at sampling)
ANCHSS: National Antenatal Sentinel HIV and Syphilis Survey Report; HIV: Human Immunodeficiency Virus; NHLS: National Health Laboratory Service; SSA: Statistics South Africa; ST: Supplementary Table.
