# Supplementary material for: It ain’t what you do, it’s the way that you do it: The pitfalls of using routine data to measure early infant HIV diagnosis in HIV-exposed infants
Source: PLoS One. 2021 Sep 30;16(9):e0257496. doi: 10.1371/journal.pone.0257496 (PMC8483382; doi:10.1371/journal.pone.0257496)
Supplement: S4 Table — (DOCX) [file pone.0257496.s004.docx]

**Supplementary Table 4: Calculation of testing coverage estimates using method 3 (NHLS-DHIS)**

| Guideline time period | Calendar year of birth | Total number of live births in facilities | Number of live births to HIV-positive women in facilities | Estimated seroprevalence | Number of live births to HIV-positive women, adjusted for births outside facilities | Number of infants tested | Number of infants tested by 7 weeks of age | **Overall testing coverage** | **Testing coverage to 7 weeks of age** | Number of infants tested  - prior to deduplication | Number of infants tested by 7 weeks of age  - prior to deduplication | **Overall testing coverage**  **- unadjusted estimate** | **Testing coverage to 7 weeks of age**  **- unadjusted estimate** |
| --- | --- | --- | --- | --- | --- | --- | --- | --- | --- | --- | --- | --- | --- |
| Source of data | | DHIS | DHIS | DHIS |  | NHLS | NHLS |  |  |  |  |  |  |
| Method of calculation | | A | B | C=B/A | D=B/0.97 | E | F | **E/D** | **F/D** | G | H | **G/B** | **H/B** |
| Prior to introduction of birth testing | April - December 2014 | 3,898 | 1,395 | 35.79% | 1,438 | 1,737 | 1,026 | **121%** | **71%** | 1,952 | 1,028 | **140%** | **74%** |
|  | January - March 2015 | 1,428 | 561 | 39.29% | 578 | 582 | 320 | **101%** | **55%** | 650 | 321 | **116%** | **57%** |
|  | Total |  | 1,965 |  | 2,016 | 2,319 | 1,346 | **115%** | **67%** | 2,602 | 1,349 | **132%** | **69%** |
| After introduction of birth testing | April - December 2015 | 3,778 | 1,475 | 39.04% | 1,521 | 2,221 | 1,463 | **146%** | **96%** | 2,678 | 1,524 | **182%** | **103%** |
|  | 2016 | 4,703 | 1,531 | 32.55% | 1,578 | 2,523 | 1,605 | **160%** | **102%** | 3,019 | 1,658 | **197%** | **108%** |
|  | Total |  | 3,006 |  | 3,099 | 4,744 | 3,068 | **153%** | **99%** | 5,697 | 3,182 | **190%** | **106%** |
| TOTAL | |  | 4,971 |  | 5,115 | 7,063 | 4,414 | **138%** | **86%** | 8,299 | 4,531 | **167%** | **91%** |

DHIS: District Health Information System; HIV: Human Immunodeficiency Virus; NHLS: National Health Laboratory Service
