# Supplementary material for: It ain’t what you do, it’s the way that you do it: The pitfalls of using routine data to measure early infant HIV diagnosis in HIV-exposed infants
Source: PLoS One. 2021 Sep 30;16(9):e0257496. doi: 10.1371/journal.pone.0257496 (PMC8483382; doi:10.1371/journal.pone.0257496)
Supplement: S5 Table — (DOCX) [file pone.0257496.s005.docx]

**Supplementary Table 5: Calculation of testing coverage estimates using method 4 (road-to-health-booklets)**

| Guideline time period | Calendar year of birth | Number of HIV-exposed infants | Number with a recorded PCR test | **Testing coverage to 7 weeks of age** |
| --- | --- | --- | --- | --- |
| Source of data | | MONARCH | MONARCH |  |
| Method of calculation | | A | B | **B/A** |
| After introduction of birth testing | July - December 2015 | 265 | 192 | **72%** |
|  | 2016 | 548 | 186 | **34%** |
| TOTAL | | 813 | 378 | **46%** |

HIV: Human Immunodeficiency Virus; MONARCH: Management and Optimization of Nutrition, Antenatal, Reproductive, Child Health and HIV Care.
